# Hypofractionated gamma knife radiosurgery for large cavernous sinus hemangiomas: Outcomes of a case series with a review of literature

**DOI:** 10.1007/s10143-026-04292-z

**Published:** 2026-04-20

**Authors:** Dogu C. Yildirim, Meryem S. Bayrakdar, Mehmet O. Askeroglu, Ali H. Duzkalir, Selcuk Peker

**Affiliations:** 1 Department of Neurosurgery, Gamma Knife Center, Koc University Hospital, Istanbul, Turkiye Turkey; 2 Faculty of Medicine, Bezmialem Vakif University, Istanbul, Turkiye Turkey; 3 Department of Neurosurgery, School of Medicine, Koc University, Istanbul, Turkiye Turkey

**Keywords:** Cavernous sinus hemangioma, Gamma knife radiosurgery, Hypofractionation, Stereotactic radiosurgery

## Abstract

Large cavernous sinus hemangiomas (CSHs) present a significant therapeutic challenge. While microsurgery carries risks of massive bleeding and morbidity, standard single-fraction stereotactic radiosurgery is often strictly limited by the radiation tolerance of the adjacent optic apparatus. Data regarding hypofractionated Gamma Knife radiosurgery (HfGKRS) for these lesions remain scarce. This study evaluates the safety and efficacy of a uniform HfGKRS protocol for large CSHs. This retrospective, single-center study analyzed four consecutive patients with large CSHs (median target volume: 25.85 cm³) treated between January 2018 and January 2025. All patients underwent mask-based HfGKRS with a standardized prescription of 25 Gy delivered in five fractions. Treatment response was assessed via volumetric MRI analysis and clinical evaluation. Toxicity was graded according to CTCAE v.5 criteria. Over a median follow-up of 30 months (range: 6–76), all tumors exhibited volumetric reduction relative to baseline, with a median shrinkage of 64% (range: 24.3%–100%). One patient achieved complete radiographic regression. Clinically, all patients demonstrated symptomatic improvement, including two complete recoveries from cranial nerve deficits. Dosimetric analysis confirmed effective sparing of critical structures; median maximum point doses to the optic apparatus (20.1 Gy) and brainstem (17.6 Gy) remained within tolerance limits. No acute or late radiation-induced toxicities were observed. HfGKRS delivering 25 Gy in five fractions appears to be a promising and feasible management strategy for large CSHs. These preliminary results suggest that this regimen can balance tumor control with the preservation of anterior visual pathways, offering a potential treatment alternative when single-fraction dosing is constrained.

## Introduction

Cavernous sinus hemangiomas (CSHs) are uncommon, benign vascular neoplasms that constitute approximately 2%–3% of all lesions in the cavernous sinus (CS), and occur more frequently in middle-aged women [7, 19]. Patients typically present with symptoms related to the mass effect within the CS, such as diplopia, facial numbness, headache, and ptosis. CSHs may attain considerable size before causing distinct neurological symptoms. Based on volumetric analysis, CSHs are classified as small (< 20 cm³), large (20–40 cm³), or giant (> 40 cm³), a criterion that plays a key role in determining the treatment strategy [23].

Diagnosis is typically established through magnetic resonance imaging (MRI), which demonstrates their characteristic radiological features [10]. Current therapeutic options include microsurgical resection, fractionated radiotherapy (RT), and stereotactic radiosurgery (SRS). Because of the risk of substantial intraoperative bleeding and postoperative morbidity associated with surgical resection, SRS and RT are generally preferred for the management of CSH [17].

In large and giant CSHs, fractionated stereotactic radiotherapy (SRT) or hypofractionated SRS is often preferred to achieve effective tumor control while preserving the integrity of surrounding critical structures such as the internal carotid artery, optic apparatus, and brainstem. This approach aims to induce long-term tumor volume reduction and prevent further growth, while minimizing radiation-induced injury to adjacent neural tissues [20].

Outcome data on hypofractionated Gamma Knife radiosurgery (HfGKRS) for large CSHs, particularly for lesions in close proximity to the optic apparatus and brainstem, remain limited in the literature. Therefore, the present study aimed to evaluate the safety and efficacy of a uniform regimen of 25 Gy delivered in five fractions for large CSHs adjacent to critical neurovascular structures.

## Materials and methods

All patients underwent detailed neurological and physical examinations, along with comprehensive radiological imaging. Treatment decisions were based on radiological diagnosis in three patients and on histopathological confirmation (biopsy) in one. No patient had undergone prior radiotherapy or SRS involving the cavernous sinus region.

During the study period, these four patients constituted the entire cohort of large CSHs (volume > 20 cm³) presenting to our Gamma Knife center. No patient with a large CSH was managed with an alternative treatment modality. The decision to use hypofractionation was driven by the intimate proximity of the tumor to the optic apparatus, which precluded safe delivery of an adequate single-fraction dose within the accepted optic pathway tolerance (≤ 8 Gy).

HfGKRS was performed using the Leksell Gamma Knife Icon™ and Esprit™ systems (Elekta Instrument AB, Stockholm, Sweden), with mask-based immobilization applied in all cases. The target volume was delineated on contrast-enhanced, 1-mm T1-weighted MRI sequences.The contrast-enhancing lesion visible on MRI was defined as the treatment target. Tumor volumes were calculated using Leksell GammaPlan (Elekta Instrument AB, Stockholm, Sweden) by manual contour delineation on consecutive slices of contrast-enhanced, 1-mm T1-weighted MRI sequences. All volumetric assessments, both pre-treatment and follow-up, were performed by a single clinician using the same software and identical MRI acquisition parameters to ensure measurement consistency.

Maximum dose constraints to critical structures were set to 25 Gy for the optic apparatus and 31 Gy for the brainstem over five fractions. These limits were established in accordance with the American Association of Physicists in Medicine Task Group 101 guidelines, aiming to optimize dose distribution while minimizing the risk of radiation-induced toxicity [2].

Because the included fractionated SRS studies used heterogeneous dose–fractionation schedules, we standardized dose comparisons using the linear–quadratic model, calculating biologically effective dose (BED) and equivalent dose in 2 Gy fractions (EQD₂). An α/β ratio of 3 Gy was assumed, consistent with the late-responding nature of CSHs.

In the absence of new symptoms, clinical and imaging follow-up was conducted six months post-treatment, and annually thereafter. Tumor volumes were measured on contrast-enhanced T1-weighted MRI, and treatment response was assessed by tumor volume change at the last imaging follow-up.

Treatment-related toxicity, including acute and late effects, was graded using the Common Terminology Criteria for Adverse Events (CTCAE), version 5 [5].

Only descriptive statistics were used due to the small sample size.

This retrospective study was approved by the Koc University Ethics Committee (IRB# 2022.022.IRB1.017), and was conducted in accordance with the World Medical Association Declaration of Helsinki.

## Results

This single-center retrospective study included four patients with large CSHs who underwent HfGKRS between January 2018 and January 2025. Three patients were female and one was male, with a median age of 38.5 years (range, 33–60). Presenting symptoms included headache (*n* = 1), ocular pain (*n* = 1), cranial nerve deficits involving the oculomotor, trochlear, and maxillomandibular branches accompanied by ptosis, anisocoria, and exophthalmos (*n* = 1), and facial numbness (*n* = 1). The median target volume was 25.85 cm³ (range, 21.4–29.9), and the median follow-up was 30 months (range, 6–76).

All patients were treated over five consecutive days with a prescribed marginal dose of 25 Gy delivered in five fractions to a median 47.5% prescription isodose line (range, 40–60). The median mean dose was 35.5 Gy (range, 31.1–38.9). Using the linear–quadratic model with an assumed α/β ratio of 3 Gy, this regimen corresponded to an estimated tumor BED₃ of 66.7 Gy and an EQD₂ of 40 Gy. On plan review, maximum point doses to the optic apparatus were 22.8, 18.3, 21.9, and 10.8 Gy, and maximum point doses to the brainstem were 18.5, 16.8, 23.6, and 11.9 Gy.

**Fig. 1:**
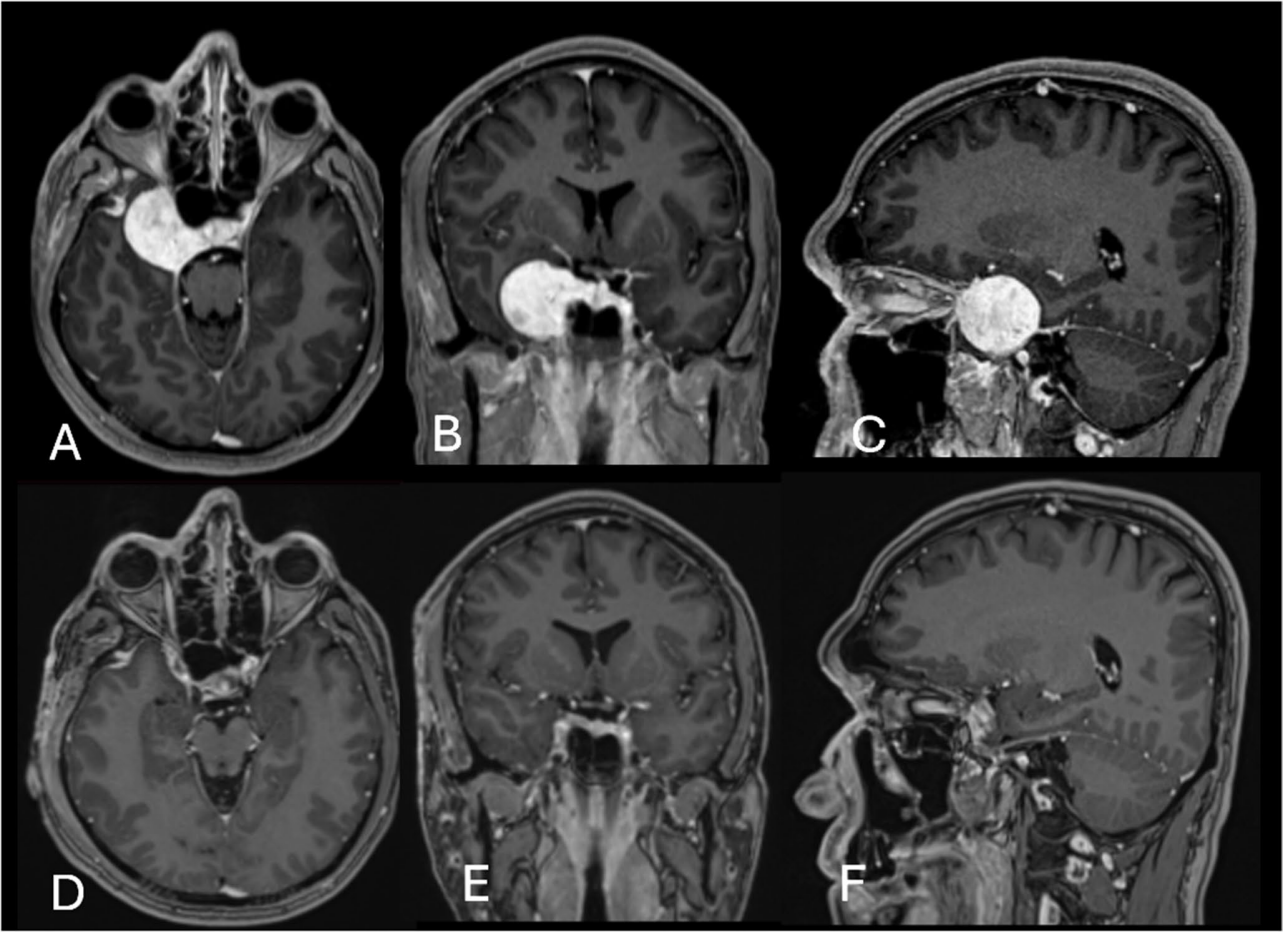
Imaging studies of Case 2 (Table 1), a 33-year-old male with a right CSH managed with HfGKRS. Axial (**A**), coronal (**B**), and sagittal (**C**) T1-weighted contrast-enhanced MR images obtained on the day of treatment, and corresponding images (D, E, and F) acquired 4 years later demonstrate complete volume reduction

All tumors exhibited volumetric reduction relative to baseline (Figs. 1 and 3). The median volume reduction was 64% (range, 24.3–100). Two patients achieved a substantial radiographic response (≥ 50% volume reduction), including one patient with complete radiographic regression.

Symptomatic improvement was first documented between 4 and 8 months after HfGKRS. Case 1 noted initial improvement at 4 months and achieved complete recovery by 12 months. Case 2 reported improvement beginning at 6 months, with complete resolution by 9 months. Case 3 demonstrated clinical improvement at the first follow-up visit (6 months); however, the 12-month follow-up had not yet been reached at the time of data analysis. Case 4 showed initial improvement at 8 months, with further progressive improvement at 12 months. No acute or late treatment-related adverse events were recorded.

Detailed patient characteristics, SRS parameters, and treatment results are provided in Table 1.

**Table 1:** Summary of 4 patients with cavernous sinus hemangioma

Case	Gender	Age	Side	Pre-GKRS operation	Symptoms at the time of GKRS	Visual field defect at the time of GKRS	Tumor volume (cm^3^)	Mean target dose (Gy)	Maximum dose received by optic pathway (Gy)	Maximum dose received by brainstem (Gy)	Follow-up (months)	Post-treatment tumor volume (cm^3^)	Volume reduction (%)	Post-GKRS toxicity	Symptom response at last follow-up	Visual field defect at last follow-up
1	F	60	L	No	Headache	No	25.1	38.9	22.8	18.5	76	2.2	91.2	No	Recovery	No
2	M	33	R	No	Ocular pain	No	26.6	36.8	18.3	16.8	48	0	100	No	Recovery	No
3	F	34	L	No	III, IV, V2-V3 CN deficits	No	21.4	34.3	21.9	23.6	6	16.2	24.3	No	Improved	No
4	F	43	R	Yes (biopsy)	Right V1-V2 numbness	No	29.9	31.1	10.8	11.9	12	18.9	36.8	No	Improved	No

All patients received a prescription (marginal) dose of 25 Gy delivered in five fractions. The mean target dose represents the average dose within the delineated target volume

## Discussion

The existing literature on fractionated or hypofractionated irradiation for CSHs predominantly involves treatments delivered using linear accelerator (LINAC)–based systems. Given the paucity of available data, only a single published report exists that presents early outcomes (6-month radiological and 9-month clinical follow-up) for two patients undergoing HfGKRS for large CSHs [19]. To our knowledge, the present study represents the first HfGKRS series for large CSHs with follow-up extending up to 76 months.

The management of CSHs involves several modalities, including microsurgical resection, conventional fractionated RT, and various forms of SRS [1]. However, the optimal treatment strategy remains a matter of debate [13]. Microsurgical resection has traditionally been employed for CSHs under the assumption that complete tumor removal could achieve a definitive cure for this benign vascular neoplasm. However, the surgical management of CSHs remains one of the most demanding challenges in neurosurgery due to the unique anatomical complexities of the CS and the marked vascularity of the tumor. Evidence from prior studies demonstrates low complete resection and elevated postoperative complication rates [12, 27]. A major risk associated with microsurgery is uncontrollable and massive intraoperative bleeding, which is often difficult to manage and can necessitate early termination of the procedure, and in severe cases may even result in death [6, 8].

CSHs are highly radiosensitive because of their vascular nature [9, 24]. Since the 1980 s, conventional fractionated RT has been used for residual or inoperable lesions and has demonstrated efficacy in inducing tumor shrinkage [15]. However, exposure of the optic apparatus or brainstem to higher doses increases the risk of radiation-induced toxicity.

Given the risks associated with microsurgical resection and the potential adverse effects of conventional RT, the role of these modalities has become increasingly limited, particularly for small and medium-sized lesions. SRT and SRS have thus emerged as preferred and often definitive treatment options [22, 23]. Due to the unique radiological features of CSH, the application of SRS as definitive treatment without the need for histopathological diagnosis has become increasingly common [4].

Regardless of the technique used (single-fraction SRS or fractionated RT), tumor control rates are universally reported as excellent or 100% in the literature [1, 14, 16, 20, 22, 23, 25]. A comprehensive meta-analysis indicated a pooled substantial radiographic response rate (tumor volume reduction > 50%) of 85% following SRS/fractionated SRS [1]. However, the application of standard, single-session SRS is severely limited when treating large (volume > 20 cm³) or giant (volume > 40 cm³) CSHs, especially when these tumors are in close proximity to the optic apparatus [22].

This limitation for large tumors has driven the adoption of fractionated or hypofractionated SRS. Fractionation leverages the radiobiological principle that sensitive normal structures can repair radiation injury during the interval between fractions, thus minimizing damage to critical structures while maintaining an effective biological dose to the target. Early investigations into hypofractionated SRS for large CSHs have primarily utilized other LINAC-based platforms, such as CyberKnife. For instance, a Phase II study involving 14 patients with large CSHs (volume > 20 cm³) treated with hypofractionated SRT (21 Gy in 3 fractions) achieved a favorable mean 77% tumor volume reduction and reported no new neurological deficits [20]. Similarly, the largest reported cohort of giant CSH (31 patients, volume > 40 cm³) treated with hypofractionated SRS (18–22 Gy in 3–4 fractions) showed a striking median tumor volume reduction of 88.1% over a median follow-up of 30 months, successfully establishing hypofractionated SRS as an effective and safe primary treatment option for giant CSHs [22].

Clinical improvement after SRS is also well documented and appears closely linked to the marked volumetric response of these tumors. Symptoms typically arise from mass effect on neurovascular structures within the CS, including cranial neuropathies, headache, and retro-orbital pain. A systematic review and meta-analysis of SRS/fractionated SRS studies (13 studies, 232 symptomatic patients) reported a pooled symptom improvement rate of 98.0% [1]. Symptom improvement can occur relatively quickly, consistent with the high radiosensitivity of CSHs [3]. In a large hypofractionated SRS series, symptomatic relief was reported 1–12 months after treatment, with complete remission of cranial neuropathies by 3 months [23]. Staged GKRS has also been associated with symptom resolution or improvement within 6 months of the first treatment [25]. Another GKRS series reported a mean time to symptom change of 3.73 months [21], and in a small cohort, complete improvement in sixth-nerve palsy was reported within one week [3]. In our series, the onset of clinical improvement ranged from 4 to 8 months, with complete recovery achieved in two patients by 9 and 12 months, respectively. These findings are in line with the reported temporal patterns in the literature [3, 22, 23] and further support the favorable clinical trajectory following hypofractionated SRS for CSHs.

The literature reviewed provides substantial evidence regarding the dosing recommendations for SRS, including single-session GKRS and fractionated/hypofractionated SRS. These recommendations are carefully balanced between achieving optimal tumor efficacy and minimizing the risk of adverse radiation effects on critical organs at risk (OAR), primarily the optic apparatus and brainstem [1, 7, 22]. For small or medium-sized CSHs that are not in close proximity to the optic pathway, single-fraction SRS is the standard approach, leveraging the tumor’s high radiosensitivity [20, 22]. Multiple series, together with an International Stereotactic Radiosurgery Society (ISRS) practice guideline, suggest a recommended single-fraction marginal dose range of 12 to 16 Gy for tumor control [1]. The clinical outcome data support this practice, with studies reporting median marginal doses around 12.6 Gy to 14.5 Gy resulting in tumor regression rates as high as 100% and substantial radiographic response rates (volume reduction ≥ 50%) pooled at 85.0% across the literature [1, 3, 11, 22].

However, the necessity of protecting the radiosensitive optic apparatus is the primary factor limiting the maximum dose delivered, especially as tumor size increases or proximity to OAR is intimate. For single-fraction SRS, maximum point doses to the optic pathways are generally constrained to 8 Gy or less, as doses between 8 Gy and 12 Gy increase the risk of radiation-induced optic neuropathy (RION), and doses exceeding 12 to 15 Gy have been associated with an estimated risk above 10% [7, 11, 18, 20, 22]. For large and giant CSHs, single-fraction SRS becomes challenging or infeasible due to these visual pathway constraints [20, 22]. Hypofractionated regimens, which are supported by ISRS, aim to deliver a total dose that is biologically comparable to established single-fraction doses while taking advantage of fractionation for normal-tissue sparing [1]. Using the linear–quadratic model with an assumed α/β of 3 Gy, representative hypofractionated schedules such as 21 Gy in 3 fractions and 22 Gy in 4 fractions correspond to BED₃ values of approximately 70 Gy and 62.3 Gy, and EQD₂ values of about 42 Gy and 37.4 Gy, respectively [20, 22]. These doses fall within the same biological range as the 25 Gy in five fractions regimen used in our series (BED₃ ≈ 66.7 Gy; EQD₂ ≈ 40 Gy), supporting the radiobiological adequacy of our protocol for large CSHs adjacent to critical structures. The maximum allowable point dose to the optic apparatus in a three-fraction regimen is approximately 17.4 Gy [20, 22]. For extremely large tumors, a staged GKRS approach (two sessions separated by several months) is also successfully implemented, with the first dose typically constrained to about 13 Gy and the second to 10 Gy, while strictly limiting the optic nerve received dose to < 9 Gy in the first stage and < 6.5 Gy in the second stage [25, 26].

While hypofractionated SRS has been successfully implemented with platforms such as CyberKnife for large and giant CSHs, HfGKRS for these lesions remains exceedingly rare. To our knowledge, only a single report describes HfGKRS as a primary treatment for large and giant CSHs [19]. In that study, two patients received 25 Gy in five fractions, and only short-term (6-month radiological and 9-month clinical follow-up) outcomes were reported. Both patients showed marked clinical improvement, including visual deterioration and headache, with changes noted as early as 3 months. This clinical success was mirrored by dramatic radiological responses: the mean tumor reduction rate was 80.4% at 6 months follow-up, with individual volume reductions reaching 85.1% and 75.6%. No new focal deficits were observed, and the only reported complication was transient temporal alopecia in one patient, which resolved by 6 months. Serial imaging did not show radiation-induced side effects at 9 months. Collectively, these findings supported the feasibility of HfGKRS in managing large-volume CSHs with meaningful and rapid volumetric reduction.

**Fig. 2:**
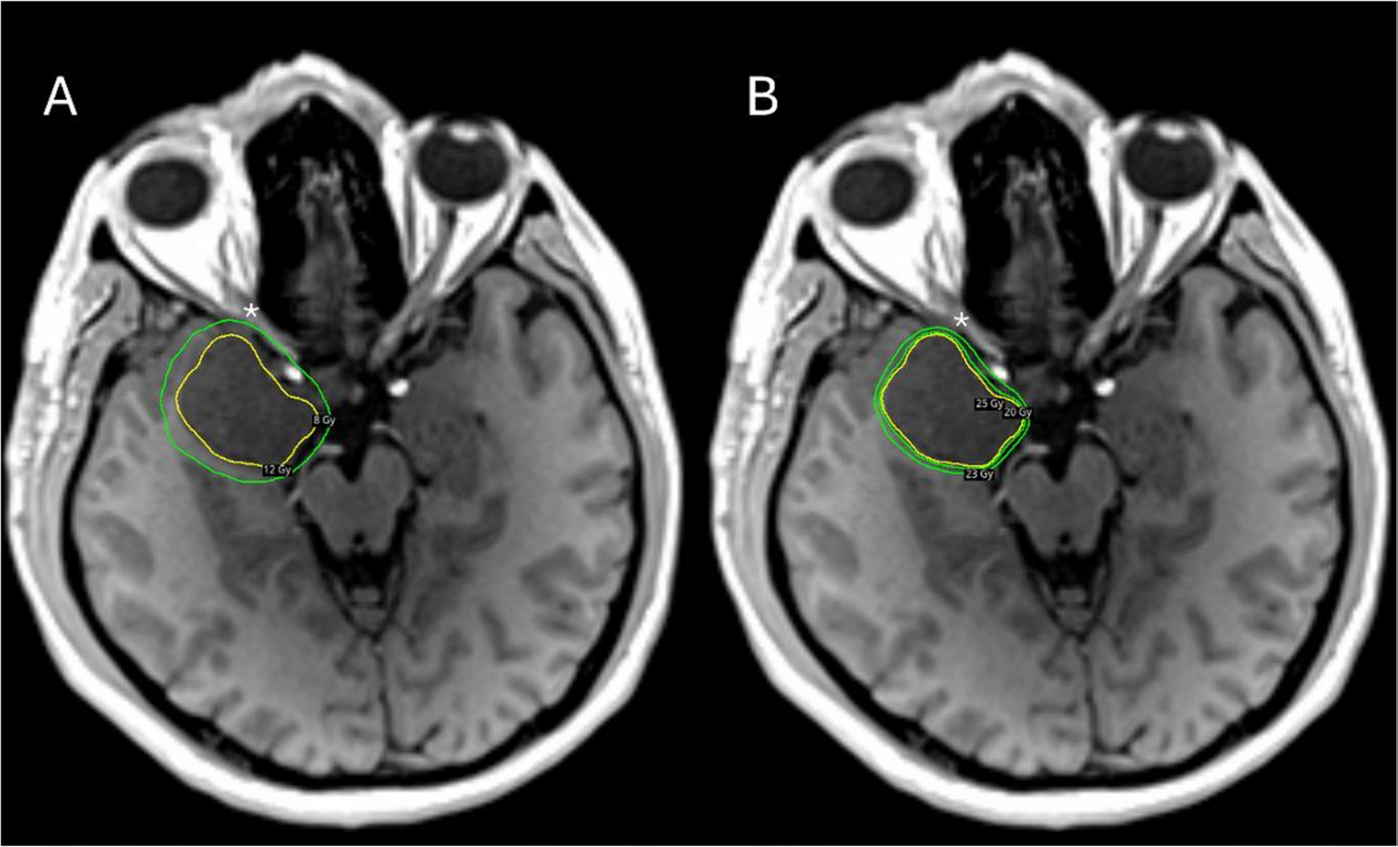
Comparative dose distribution on axial non-contrast T1-weighted MRI (selected for optimal optic nerve visualization) in a representative case (Case 2). (**A**) Hypothetical single-fraction plan delivering 12 Gy marginal dose: the 8 Gy isodose line (green), which represents the widely accepted single-fraction tolerance threshold for the optic apparatus (asterisk), extends to encompass the ipsilateral optic nerve, indicating that safe single-fraction treatment would not be feasible for this large, optic-adjacent lesion. (**B**) Actual hypofractionated plan delivering 25 Gy in five fractions: the prescription isodose (25 Gy, yellow) provides conformal target coverage, while the 23 Gy and 20 Gy (green, outer) isodose lines remain well clear of the optic nerve (asterisk), demonstrating effective sparing of the anterior visual pathway. The five-fraction tolerance limit for the optic apparatus (25 Gy) is not reached at the optic nerve in this plan, confirming the dosimetric advantage of the hypofractionated approach

In our consecutive four-patient series of large CSHs, we treated all lesions with a uniform HfGKRS regimen (25 Gy in 5 fractions). All lesions regressed radiographically (median reduction 64%; including one complete response), and all patients improved clinically (two complete recoveries and two partial improvements) without recorded acute or late toxicity over a median 30-month follow-up (range, 6–76 months). Median maximum point doses for optic apparatus (20.1 Gy) and brainstem (17.6 Gy) remained within widely used five-fraction tolerance ranges, illustrating that tumor-directed BED can be maintained while respecting visual pathway and brainstem constraints in large, optic-adjacent lesions. The steep dose gradient achieved at the tumor–optic apparatus interface is illustrated in a representative treatment plan (Fig. 2).

**Fig. 3:**
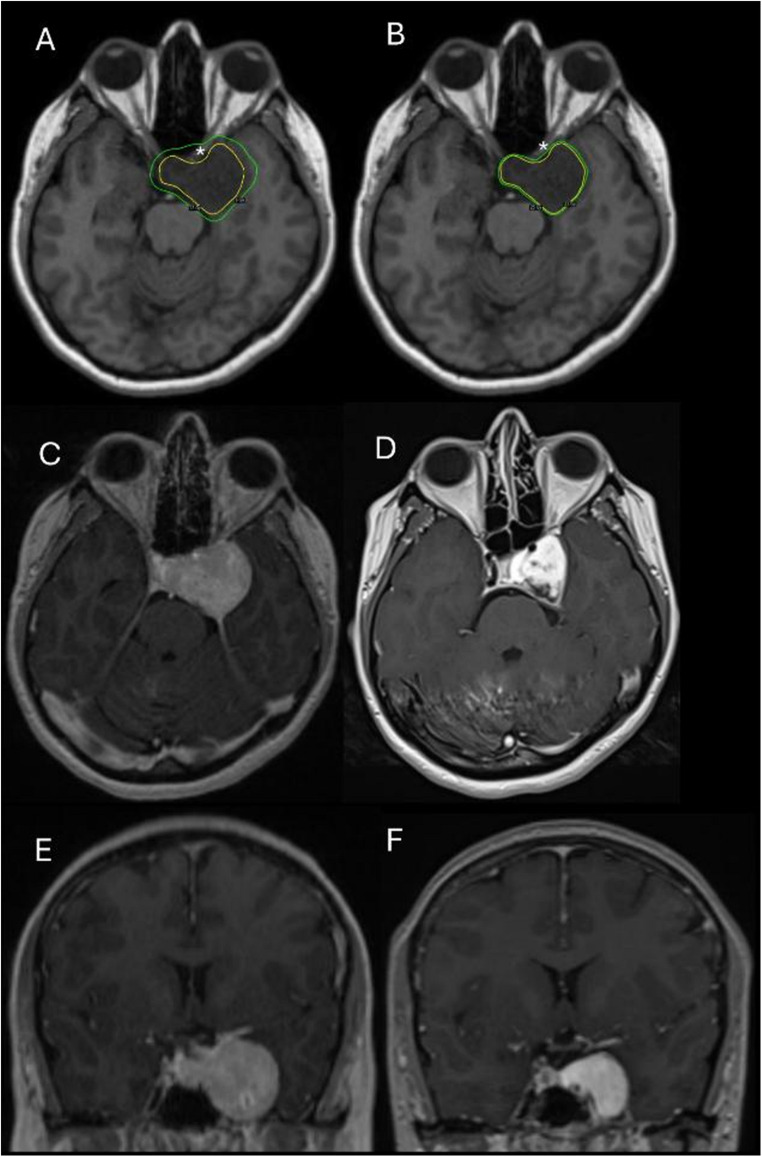
Representative imaging and treatment planning of Case 3 (Table 1), a 34-year-old female with a left CSH (21.4 cm³) presenting with CN III, IV, and V2–V3 deficits. (**A**) Hypothetical single-fraction plan delivering 12 Gy marginal dose (yellow) on axial non-contrast T1-weighted MRI: the 8 Gy isodose line (green), representing the single-fraction optic apparatus tolerance threshold, encompasses the left optic nerve (asterisk), precluding safe single-fraction treatment. (**B**) Actual hypofractionated plan delivering 25 Gy in five fractions: the prescription isodose (25 Gy, yellow) provides conformal target coverage, confirming adequate sparing of left optic nerve (asterisk). Axial (**C**) and coronal (**E**) contrast-enhanced T1-weighted MRI obtained on the day of treatment demonstrate a large left cavernous sinus mass. Corresponding axial (**D**) and coronal (**F**) contrast-enhanced T1-weighted MRI at 6-month follow-up show volumetric reduction of 24.3%

Compared with the predominantly LINAC-based hypofractionation literature, our principal contribution is the use of a single standardized 25 Gy in five fractions protocol delivered on Gamma Knife platforms, with consistent dosimetry and follow-up extending to 76 months. These preliminary results suggest that HfGKRS may be a practical and safe option when single-fraction dosing is constrained by proximity to critical structures (Fig. 3).

## Limitations

This investigation is a single-center, retrospective case series with a small cohort size (*n* = 4), which inherently limits the statistical power and reduces the external validity of the findings. The small sample also restricts the ability to detect less common or rare adverse events that may be associated with the treatment protocol.

Furthermore, follow-up duration was heterogeneous across the cohort. While one patient was followed for 76 months, two patients (Cases 3 and 4) had follow-up limited to 6 and 12 months, respectively. As late radiation-induced complications may take years to manifest, the safety profile for these patients reflects only early outcomes, and continued surveillance is necessary.

Additionally, serial volumetric measurements at interim follow-up time points were not systematically performed in all cases. Therefore, the kinetics of tumor regression could not be characterized, and only pre-treatment and last follow-up volumes are reported.

In three of the four cases, management was based on characteristic MRI findings without histopathologic confirmation. Although the radiologic phenotype of CSH is well described, this approach introduces a risk of diagnostic misclassification.

Moreover, the dosimetric findings may not be broadly generalizable, as all patients were treated with the same mask-based HfGKRS regimen (25 Gy in five fractions) using center-specific planning parameters.

## Conclusion

A uniform, mask-based HfGKRS protocol for large CSHs achieved radiographic regression and clinical improvement in all patients, with no observed toxicity and adherence to predefined dose constraints for the optic apparatus and brainstem. By focusing specifically on large, optic apparatus adjacent lesions and using a single standardized regimen on GKRS with follow-up extending to 76 months, this series adds dedicated GKRS-based evidence to a literature largely dominated by LINAC-based hypofractionation. These preliminary findings suggest that HfGKRS may represent a feasible and safe treatment option when single-fraction dosing is constrained by critical structures, and they provide an initial planning framework for centers managing challenging CSHs in critical neurovascular territory. Further studies with larger cohorts and longer follow-up are warranted to validate these results.

## Data Availability

No datasets were generated or analysed during the current study.
